# Neutrophil-to-lymphocyte ratio as a prognostic factor for patients with metastatic or recurrent breast cancer treated using capecitabine: a retrospective study

**DOI:** 10.1186/s12885-021-09112-9

**Published:** 2022-01-14

**Authors:** Shigemasa Takamizawa, Tatsunori Shimoi, Natsuko Satomi-Tsushita, Shu Yazaki, Toshihiro Okuya, Yuki Kojima, Hitomi Sumiyoshi-Okuma, Tadaaki Nishikawa, Maki Tanioka, Kazuki Sudo, Emi Noguchi, Kan Yonemori

**Affiliations:** Department of Medical Oncology, National Cancer Center Hospital, 5-1-1 Tsukiji, Chuo-Ku, 104-0045 Tokyo, Japan

**Keywords:** Breast cancer, Capecitabine, Chemotherapy, Eribulin, Neutrophil-to-lymphocyte ratio, NLR, Prognostic factor

## Abstract

**Background:**

Eribulin or capecitabine monotherapy is the next cytotoxic chemotherapy option for patients with metastatic or recurrent breast cancer who have previously received an anthracycline or a taxane. However, it is unclear what factors can guide the selection of eribulin or capecitabine in this setting, and prognostic factors are needed to guide appropriate treatment selection. The neutrophil-to-lymphocyte ratio (NLR) is a prognostic factor for eribulin-treated patients, although it is unclear whether it is a prognostic factor for capecitabine-treated patients. Therefore, we analysed the ability of the NLR to predict oncological outcomes among patients who received capecitabine after previous anthracycline or taxane treatment for breast cancer.

**Methods:**

We retrospectively reviewed the medical records of patients with metastatic or recurrent breast cancer who had previously received anthracycline or taxane treatment at the National Cancer Center Hospital between 2007 and 2015. Patients were included if they received eribulin or capecitabine monotherapy as first-line, second-line, or third-line chemotherapy. Analyses of overall survival (OS) and progression-free survival (PFS) were performed according to various factors.

**Results:**

Between 2007 and 2015, we identified 125 eligible patients, including 46 patients who received only eribulin, 34 patients who received only capecitabine, and 45 patients who received eribulin and capecitabine. The median follow-up period was 19.1 months. Among eribulin-treated patients, an NLR of <3 independently predicted better OS. Among capecitabine-treated patients, an NLR of <3 independently predicted better PFS but not better OS. In addition, a lymphocyte-to-monocyte ratio of ≥5 was associated with better PFS and OS.

**Conclusions:**

To the best of our knowledge, this is the first study to evaluate whether the NLR is a prognostic factor for capecitabine-treated patients with metastatic or recurrent breast cancer. However, the NLR only independently predicted PFS in this setting, despite it being a useful prognostic factor for other chemotherapies.

**Supplementary Information:**

The online version contains supplementary material available at 10.1186/s12885-021-09112-9.

## Background

Breast cancer is the most common malignancy among women worldwide [1], and patients with metastatic or recurrent human epidermal growth factor 2 (HER2)-negative breast cancer have a poor prognosis, especially if they have previously received anthracycline or taxane treatment. The EMBRACE trial revealed that eribulin provided an improvement in overall survival (OS), relative to the physician’s choice of treatment, in patients with metastatic or recurrent breast cancer [2]. However, another phase III study (Study 301) revealed that eribulin was not superior to capecitabine in terms of OS or progression-free survival (PFS) in this setting [3]. Thus, eribulin monotherapy or capecitabine monotherapy has become the most common real-world cytotoxic chemotherapy for patients who were previously treated using anthracycline or taxane [4], although no standard chemotherapy has been established for these patients if they do not have *BRCA* loss-of-function mutations. Additional treatment options for these patients include vinorelbine or gemcitabine monotherapy.

Effective prognostic factors are needed to guide the selection of appropriate treatment for breast cancer, and reported prognostic factors include tumour size, stage, histological grade, lymph node status, hormone receptor (HR) status, and age [5]. However, these factors are typically used to predict the prognosis of patients with resectable breast cancer and thus are often not useful for guiding the selection of cytotoxic chemotherapy for metastatic or recurrent breast cancer. Thymidine phosphorylase expression has been reported as a biomarker of sensitivity to capecitabine treatment [6] and a predictive marker of docetaxel-modulated capecitabine treatment for patients with metastatic breast cancer [7]. However, using thymidine phosphorylase for guiding the appropriate treatment is not a straightforward approach as the expression is measured by immunohistochemistry of paraffin-embedded cancer tissues. Serum microRNA profiling is reportedly a biomarker for the effectiveness of eribulin and the development of new distant metastases in cases of metastatic breast cancer [8], although this biomarker is difficult to measure in a clinical setting.

Inflammation is a critical factor in tumour development and progression [9]. Thus, various studies have evaluated whether the prognosis of patients with breast cancer and other malignancies can be predicted using systemic inflammatory markers, such as lactate dehydrogenase (LDH) [10], C-reactive protein (CRP) [11, 12], albumin [13], the platelet-to-lymphocyte ratio (PLR) [14], absolute lymphocyte count (ALC) [15, 16], and the lymphocyte-to-monocyte ratio (LMR) [17–19]. The neutrophil-to-lymphocyte ratio (NLR) in peripheral blood, which is a marker of systemic immunity and inflammation, is also reportedly able to predict the prognosis of patients with solid tumours [12, 20] and breast cancer [21]. NLR has also been reported as a prognostic factor for patients with metastatic breast cancer [22]. Furthermore, relative to other chemotherapies, eribulin may play a relatively greater role in the relationship between the NLR and prognosis, as a low baseline NLR was significantly associated with improved outcomes among patients who received eribulin for locally advanced or metastatic breast cancer [23]. The NLR can predict outcomes among patients who received eribulin for metastatic breast cancer [24], and the NLR may be a more general prognostic factor, rather than a specific predictor of eribulin efficacy [16]. As the NLR is easily, rapidly, and readily determined using peripheral blood samples, it might be useful for guiding treatment for patients in clinical practice if it is confirmed to have prognostic value.

We are not aware of any reports regarding whether the NLR can predict outcomes among patients who receive capecitabine for metastatic or recurrent breast cancer. In addition, eribulin monotherapy or capecitabine monotherapy is the next cytotoxic chemotherapy option for breast cancer patients who have previously received anthracycline or taxane treatment, although it is unclear how to select the most appropriate option in this setting. Therefore, the present study aimed to evaluate whether the prognostic value of the NLR varies according to the use of eribulin or capecitabine, which could help guide treatment selection among patients who are eligible to receive eribulin monotherapy or capecitabine monotherapy for metastatic or recurrent breast cancer.

## Methods

### Study cohort

We retrospectively reviewed the medical records of patients with metastatic or recurrent breast cancer who had previously received anthracycline or taxane treatment at the National Cancer Center Hospital between 2007 and 2015. Patients with any type of simultaneous metastatic cancer were excluded. Patients were considered eligible if they had been treated using eribulin or capecitabine monotherapy as a first-line, second-line, or third-line chemotherapy for metastatic or recurrent breast cancer. Eribulin or capecitabine treatment was continued until tumour progression or the appearance of severe adverse events. The retrospective study protocol was approved by the institutional review board of the National Cancer Center Hospital (NCCH 2014-092) and complied with the Declaration of Helsinki.

### Variables

Immunohistochemical staining at the time of the pathological diagnosis was performed to determine each patient’s oestrogen receptor (ER), progesterone receptor (PgR), and HER2 statuses. Histopathological grading and immunohistochemical staining results for ER, PgR, and HER2 were interpreted based on previously reported guidelines [25]. Performance status (PS) was evaluated according to the Eastern Cooperative Oncology Group (ECOG) criteria. Tumour responses were assessed by the investigators according to the Response Evaluation Criteria in Solid Tumors (version 1.1). The overall response rate (ORR) was defined as the proportion of patients who achieved a complete response (CR) or partial response (PR). The OS interval was calculated from the start of eribulin or capecitabine treatment until death because of any cause or censoring at the last date of confirmed survival. The PFS interval was calculated from the start of treatment until the first instance of disease progression, death because of any cause, or censoring at the last date of confirmed survival without disease progression.

### Blood sample analysis

Blood sample data were eligible for analysis if performed within 7 days before the start of eribulin or capecitabine monotherapy. NLR, LMR, and PLR were defined as the absolute neutrophil count divided by the absolute lymphocyte count, the absolute lymphocyte count divided by the absolute monocyte count, and the absolute platelet count divided by the absolute lymphocyte count, respectively.

### Statistical analysis

Univariate analyses were performed to determine whether the ORR, OS, and PFS were associated with receptor status, surgical history, treatment history, albumin concentration (cut-off: 4.1 g/dL), age (cut-off: 60 years), LDH concentration (cut-off: 222 U/L), CRP concentration (cut-off: 0.15 mg/dL), NLR (cut-off: 3), ALC (cut-off: 1,500/µL), LMR (cut-off: 5), and PLR (cut-off: 250). These cut-off values were selected based on previous reports [10–19]. Multivariable Cox proportional hazard models were used to analyse OS and PFS in two scenarios: (A) when the NLR was a definitive prognostic factor and using age, HR status, HER2 status, and NLR or (B) when the inflammatory markers that predicted PFS in the univariate analyses were also included (age, HR status, HER2 status, ALC, NLR, LMR, and PLR). The ORR was analysed using the chi-squared test, while the OS and PFS outcomes were analysed using the Wilcoxon signed-rank sum test. Survival curves were also created using the Kaplan–Meier method. All statistical analyses were performed using JMP software (version 14.3.0 for Windows; SAS Institute Japan Inc., Cary, NC, USA), and results were considered statistically significant at a two-sided *p*-value of <0.05.

## Results

### Patient characteristics

Between 2007 and 2015, we identified 125 patients who received eribulin and/or capecitabine for metastatic or recurrent breast cancer and had previously received an anthracycline or a taxane. The first-line, second-line, or third-line treatments for metastatic or recurrent breast cancer involved only eribulin (46 patients), only capecitabine (34 patients), or both eribulin and capecitabine (45 patients). All patients were female, and the median ages were 56 years (range: 30–76 years) for patients who received eribulin monotherapy and 59 years (range: 36–74 years) for patients who received capecitabine monotherapy. The patient characteristics are shown in Table 1 and Additional file 1. Relative to patients who received eribulin, patients who received capecitabine had a significantly lower LDH concentration and a significantly higher LMR. Capecitabine was administered at a significantly earlier line than eribulin, and patients who received capecitabine monotherapy had a significantly better response than patients who received eribulin monotherapy. Hormone therapy was significantly more often administered before chemotherapy for patients who received capecitabine than patients who received eribulin. The median follow-up period was 19.1 months.

**Table 1:** Patient characteristics

		Eribulin (*n* = 91)	Capecitabine (*n* = 79)	*p*
Age in years, n (%)	Median (range)	56 (30–76)	59 (36–74)	-
	≥60	34 (37)	35 (44)	0.43
	<60	57 (63)	44 (56)	
Sex, n (%)	Male	0 (0)	0 (0)	-
	Female	91 (100)	79 (100)	
ECOG-PS, n (%)	0	55 (60)	43 (54)	0.44
	1	36 (40)	35 (44)	
	2	0 (0)	1 (1)	
HR, n (%)	Positive	71 (78)	70 (89)	0.1
	Negative	20 (22)	9 (11)	
ER, n (%)	Positive	68 (75)	65 (82)	0.27
	Negative	23 (25)	14 (18)	
PgR, n (%)	Positive	55 (60)	52 (66)	0.42
	Negative	36 (40)	25 (32)	
	NA	0 (0)	2 (3)	-
HER2, n (%)	Positive	5 (6)	2 (3)	0.45
	Negative	85 (93)	75 (95)	
	NA	1 (1)	2 (3)	-
Triple-negative, n (%)		17 (19)	9 (11)	0.21
Surgical history, n (%)	Positive	83 (91)	72 (91)	1.0
	Negative	8 (9)	7 (9)	
Neoadjuvant/adjuvant chemotherapy, n (%)	Positive	76 (84)	63 (80)	0.56
	Negative	15 (16)	16 (20)	
Previous hormone therapy, n (%)	Positive	63 (69)	72 (91)	**0.001**
	Negative	28 (31)	7 (9)	
Previous anthracycline, n (%)	Positive	85 (93)	67 (85)	0.08
	Negative	6 (7)	12 (15)	
Previous taxane, n (%)	Positive	88 (97)	73 (92)	0.31
	Negative	3 (3)	6 (8)	
Previous chemotherapy regimens, n (%)	0	11 (12)	18 (23)	**0.001**
	1	29 (32)	39 (49)	
	2	51 (56)	22 (28)	
Response, n (%)	PR	14 (15)	16 (20)	**0.018**
	SD	43 (47)	49 (62)	
	PD	34 (37)	14 (18)	
Albumin, n (%)	≥4.1 g/dL	55 (60)	51 (65)	0.64
	<4.1 g/dL	36 (40)	28 (35)	
LDH, n (%)	<222 U/L	38 (42)	48 (61)	**0.01**
	≥222 U/L	53 (58)	31 (39)	
CRP, n (%)	<0.15 mg/dL	39 (43)	46 (58)	0.06
	≥0.15 mg/dL	52 (57)	33 (42)	
NLR, n (%)	<3	65 (71)	56 (71)	1.0
	≥3	26 (29)	23 (29)	
ALC, n (%)	≥1,500/µL	31 (34)	36 (46)	0.16
	<1,500/µL	60 (66)	43 (54)	
LMR, n (%)	≥5	36 (40)	45 (57)	**0.03**
	<5	55 (60)	34 (43)	
PLR, n (%)	<250	73 (80)	68 (86)	0.41
	≥250	18 (20)	11 (14)	

*ALC* absolute lymphocyte count; *CRP* C-reactive protein; *ECOG-PS* Eastern Cooperative Oncology Group-performance status; *ER* oestrogen receptor; *HER2* human epidermal growth factor receptor 2; *HR* hormone receptor; *LDH* lactate dehydrogenase; *LMR* lymphocyte-to-monocyte ratio; *NA* not available; *NLR* neutrophil-to-lymphocyte ratio; *PD* progressive disease; *PgR* progesterone receptor; *PLR* platelet-to-lymphocyte ratio; *PR* partial response; *SD* stable disease

### PFS and OS after starting eribulin or capecitabine monotherapy

**Fig. 1:**
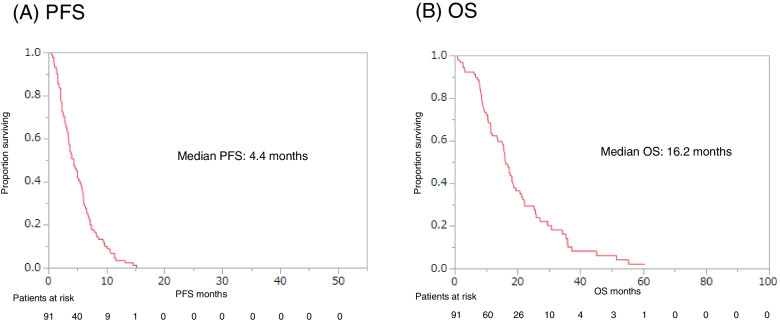
Progression-free survival (PFS, **A**) and overall survival (OS, **B**) starting from first day of eribulin monotherapy

**Fig. 2:**
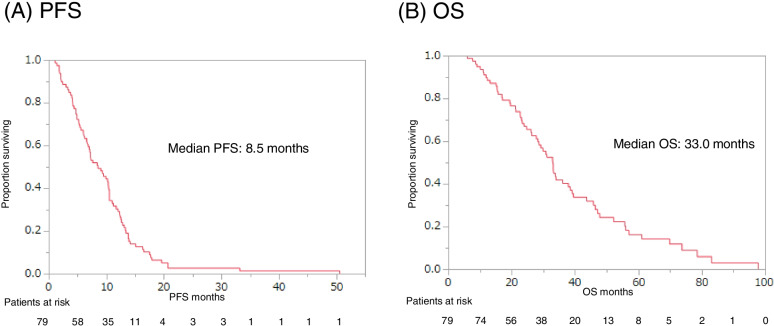
Progression-free survival (PFS, **A**) and overall survival (OS, **B**) starting from first day of capecitabine monotherapy

**Fig. 3:**
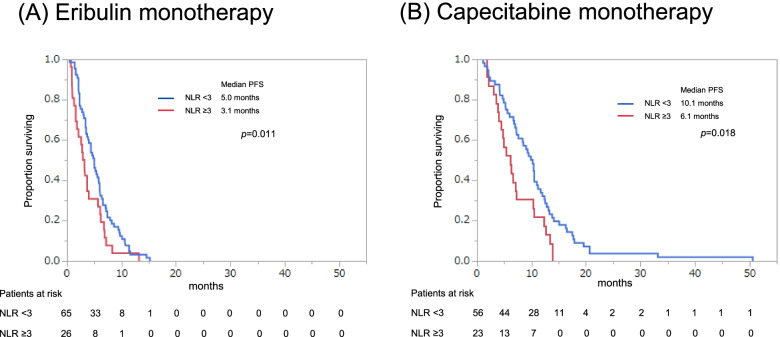
Progression-free survival (PFS) stratified according to the neutrophil-to-lymphocyte ratio (NLR, (<3 vs. ≥3) starting from first day of eribulin monotherapy (**A**) or capecitabine monotherapy (**B**)

**Fig. 4:**
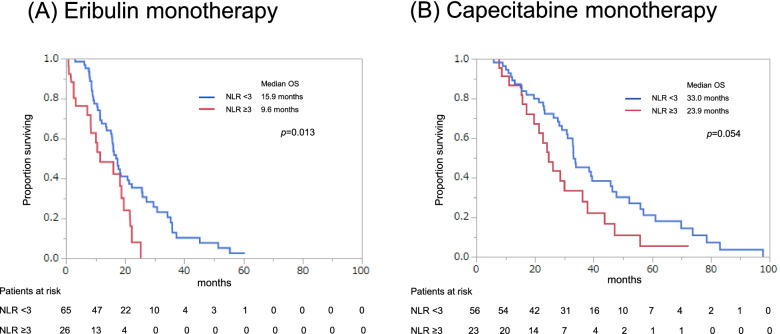
Overall survival (OS) stratified according to the neutrophil-to-lymphocyte ratio (NLR, (<3 vs. ≥3) starting from first day of eribulin monotherapy (**A**) or capecitabine monotherapy (**B**)

Eribulin was administered to 91 patients, with or without capecitabine monotherapy, and these patients had a median PFS of 4.4 months and a median OS of 16.2 months (Fig. 1). Capecitabine was administered to 79 patients, with or without eribulin monotherapy, and these patients had a median PFS of 8.5 months and OS of 33.0 months (Fig. 2). The PFS and OS curves stratified according to NLR (<3 vs. ≥3) revealed that a lower NLR was associated with significantly better outcomes in the eribulin and capecitabine groups (Figs. 3 and 4).

### Univariate analyses of ORR

The univariate analyses revealed that, among patients who received eribulin monotherapy, a significantly better ORR was associated with HR+ status (*p*=0.034) and ER+ status (*p*=0.018). Furthermore, among patients who received capecitabine monotherapy, significantly better ORR was associated with an NLR of <3 (*p*=0.03) (Additional file 2).

### Univariate analyses of PFS

The univariate analyses revealed that, among patients who received eribulin monotherapy, significantly better PFS was associated with an NLR <3 (*p*=0.011), a PLR of <250 (*p*=0.001), a surgical history (*p*=0.006), and neoadjuvant/adjuvant chemotherapy (*p*=0.045). Among patients who received capecitabine monotherapy, significantly better PFS was associated with an NLR of <3 (*p*=0.011), ER+ status (*p*=0.029), a surgical history (*p*=0.036), an ALC of ≥1,500/µL (*p*=0.013), an LMR of ≥5 (*p*=0.001), and a PLR of <250 (*p*=0.037) (Additional file 3).

### Multivariable analyses of PFS

When the NLR was treated as a definitive prognostic factor, an NLR of <3 predicted significantly better PFS among patients who received capecitabine monotherapy (*p*=0.01) but not among patients who received eribulin monotherapy (Table 2). When inflammatory markers that predicted PFS were also included in the multivariable model, better PFS was predicted by an LMR of ≥5 among patients who received capecitabine monotherapy (*p*=0.03) and a PLR of <250 among patients who received eribulin monotherapy (*p*=0.005) (Table 2).

**Table 2:** Multivariable analyses of progression-free survival

	Eribulin (n = 91)		Capecitabine (n = 79)	
	Hazard ratio (95% CI)	*p*	Hazard ratio (95% CI)	*p*
**When the NLR was a definitive prognostic factor**				
Age (≥60 years vs. <60 years)	0.90 (0.57–1.38)	0.62	0.92 (0.56–1.48)	0.72
HR (positive vs. negative)	0.91 (0.54–1.62)	0.74	1.22 (0.62–2.71)	0.58
HER2 (negative vs. positive)	0.72 (0.30–2.15)	0.52	0.90 (0.27–5.60)	0.89
NLR (<3 vs. ≥3)	0.62 (0.39–1.01)	0.05	**0.48 (0.29–0.84)**	**0.01**
**When inflammatory markers that predicted PFS were also included**				
Age (≥60 years vs. <60 years)	0.82 (0.51–1.31)	0.42	0.80 (0.48–1.31)	0.38
HR (positive vs. negative)	0.94 (0.55–1.70)	0.83	1.26 (0.59–2.97)	0.57
HER2 (negative vs. positive)	0.93 (0.37–2.88)	0.89	1.64 (0.44–10.7)	0.49
ALC (≥1,500/µL vs. <1,500/µL)	0.96 (0.57–1.61)	0.89	0.68 (0.38–1.22)	0.19
NLR (<3 vs. ≥3)	0.69 (0.41–1.20)	0.18	0.86 (0.40–1.91)	0.70
LMR (≥5 vs. <5)	1.20 (0.73–1.97)	0.47	**0.55 (0.33–0.94)**	**0.03**
PLR (<250 vs. ≥250)	**0.40 (0.22–0.75)**	**0.005**	0.79 (0.32–1.99)	0.62

*ALC* absolute lymphocyte count; *CI* confidence interval; *CRP* C-reactive protein; *ER* oestrogen receptor; *HER2* human epidermal growth factor receptor 2; *HR* hormone receptor; *LDH* lactate dehydrogenase; *LMR* lymphocyte-to-monocyte ratio; *NLR* neutrophil-to-lymphocyte ratio; *PFS* progression-free survival; *PgR* progesterone receptor; *PLR* platelet-to-lymphocyte ratio

### Univariable analyses of OS

The univariate analyses revealed that, among patients who received eribulin monotherapy, significantly better OS was associated with HR+ status (*p*=0.013), PgR+ status (*p*=0.04), a surgical history (*p*=0.024), previous hormone therapy (*p*=0.01), an LDH concentration of <222 U/L (*p*=0.001), an NLR of <3 (*p*=0.013), an LMR of ≥5 (*p*=0.013), and a PLR of <250 (*p*=0.002). Among patients who received capecitabine monotherapy, significantly better OS was associated with an LDH concentration of <222 U/L (*p*=0.002), a CRP concentration of <0.15 mg/dL (*p*=0.019), an NLR of <3 (*p*=0.037), and an LMR of ≥5 (*p*=0.014) (Table 3).

**Table 3:** Univariate analyses of overall survival

	Eribulin (n = 91)	Capecitabine (n = 79)
	*p*	*p*
HR+	**0.013**	0.39
ER+	0.13	0.05
PgR+	**0.04**	0.84
HER2-	0.84	0.89
Surgical history	**0.024**	0.85
Neoadjuvant/adjuvant chemotherapy	0.63	0.58
Previous hormone therapy	**0.01**	0.8
Previous anthracycline	0.35	0.09
Previous taxane	0.13	0.28
Albumin ≥4.1 g/dL	0.06	0.12
Age ≥60 years	0.12	0.63
LDH <222 U/L	**0.001**	**0.002**
CRP <0.15 mg/dL	0.09	**0.019**
NLR <3	**0.013**	**0.037**
ALC ≥1,500/µL	0.11	0.06
LMR ≥5	**0.013**	**0.014**
PLR <250	**0.002**	0.13

*ALC* absolute lymphocyte count; *CRP* C-reactive protein; *ER* oestrogen receptor; *HER2* human epidermal growth factor receptor type 2; *HR* hormone receptor; *LDH* lactate dehydrogenase; *LMR* lymphocyte-to-monocyte ratio; *NLR* neutrophil-to-lymphocyte ratio; *PgR* progesterone receptor; *PLR* platelet-to-lymphocyte ratio

### Multivariable analyses of OS

When the NLR was treated as a definitive prognostic factor, an NLR of <3 predicted significantly better OS among patients who received eribulin monotherapy (*p*=0.03) but not among patients who received capecitabine monotherapy (Table 4). When inflammatory markers that predicted PFS were also included in the multivariable model, better OS was predicted by an LMR of ≥5 among patients who received capecitabine monotherapy (*p*=0.03) (Table 4).

**Table 4:** Multivariable analyses of overall survival

	Eribulin (*n* = 91)		Capecitabine (*n* = 79)	
	Hazard ratio (95% CI)	*p*	Hazard ratio (95% CI)	*p*
**When the NLR was a definitive prognostic factor**				
Age (≥60 years vs. <60 years)	0.74 (0.44–1.21)	0.24	0.92 (0.53–1.56)	0.76
HR (positive vs. negative)	0.57 (0.32–1.06)	0.08	1.05 (0.52–2.42)	0.89
HER2 (negative vs. positive)	0.87 (0.25–5.44)	0.85	1.08 (0.23–19.3)	0.94
NLR (<3 vs. ≥3)	**0.53 (0.30–0.95)**	**0.03**	0.56 (0.32–1.01)	0.05
**When inflammatory markers that predicted PFS were also included**				
Age (≥60 years vs. <60 years)	0.60 (0.35–1.01)	0.06	0.74 (0.41–1.30)	0.3
HR (positive vs. negative)	0.54 (0.30–1.01)	0.06	1.60 (0.73–3.91)	0.25
HER2 (negative vs. positive)	1.05 (0.30–6.69)	0.94	2.34 (0.45–43.1)	0.36
ALC (≥1,500/μL vs. <1,500/μL)	0.72 (0.37–1.40)	0.33	0.57 (0.28–1.18)	0.13
NLR (<3 vs. ≥3)	0.80 (0.42–1.56)	0.51	1.65 (0.61–4.63)	0.32
LMR (≥5 vs. <5)	0.64 (0.35–1.18)	0.15	**0.50 (0.27–0.94)**	**0.03**
PLR (<250 vs. ≥250)	0.63 (0.33–1.30)	0.2	0.50 (0.18–1.35)	0.17

*ALC* absolute lymphocyte count; *CRP* C-reactive protein; *ER* oestrogen receptor; *HER2* human epidermal growth factor receptor 2; *HR* hormone receptor; *LDH* lactate dehydrogenase; *LMR* lymphocyte-to-monocyte ratio; *NLR* neutrophil-to-lymphocyte ratio; *PFS* progression-free survival; *PgR* progesterone receptor; *PLR* platelet-to-lymphocyte ratio

## Discussion

This retrospective study evaluated whether the NLR could predict oncological outcomes after eribulin or capecitabine monotherapy for patients with metastatic or recurrent breast cancer who had previously received an anthracycline or a taxane. The multivariable analysis revealed that an NLR of <3 predicted significantly better OS among patients who received eribulin, which agrees with previously reported results [23, 24]. However, among patients who received capecitabine, an NLR of <3 independently predicted better PFS but not better OS. Therefore, we cannot conclude that the NLR is useful for predicting OS among patients who receive capecitabine for metastatic or recurrent breast cancer. We also evaluated various other potential prognostic factors, which revealed that a PLR of <250 predicted better PFS among patients who received eribulin monotherapy, while an LMR of ≥5 predicted better PFS and OS among patients who received capecitabine monotherapy.

Effective prognostic factors, including thymidine phosphorylase and serum microRNA, have been identified to guide the selection of appropriate treatment for metastatic breast cancer [6–8]. In addition, NLR has been predicted to be a prognostic factor in clinical practice. Despite the ability of the NLR to predict outcomes after other chemotherapies, we did not observe an independent association between the NLR and OS among patients who received capecitabine for metastatic or recurrent breast cancer. This lack of an association may be related to the patients’ characteristics and the NLR itself. For example, capecitabine was administered at a significantly earlier line than eribulin, and >70% of patients received capecitabine as first-line or second-line treatment. Thus, the relationship between NLR and OS after capecitabine treatment might have been weakened by the effects of subsequent treatment lines. In addition, the NLR might only be a useful prognostic factor for patients with ER-negative and HER2-negative breast cancer [21], while the present study included patients with various HR subtypes of breast cancer. Furthermore, the NLR might not accurately reflect the patient’s inflammatory status in this setting, as chemotherapy-induced myelosuppression can lead to neutropenia. In contrast, platelets or monocytes are less sensitive to myelosuppression (vs. neutrophils). Thus, the PLR or LMR might be more accurate inflammatory markers and better able to predict survival outcomes in this setting. Moreover, overexpression of immune-related genes might not predict the response to capecitabine monotherapy [26], which could suggest that the NLR (as an inflammatory marker) might not be an appropriate prognostic factor for capecitabine-treated patients. Further studies are needed to identify more appropriate non-inflammatory markers that can be used to predict survival outcomes after starting capecitabine monotherapy in this setting.

The present study has three important limitations. First, the sample size was small, which could limit the power of the analyses. Second, many cases were missing information regarding the histological grade (which is generally considered a prognostic factor), and we were unable to consider this variable in the analyses. Finally, we were unable to evaluate prognostic factors such as thymidine phosphorylase and serum microRNA.

## Conclusions

To the best of our knowledge, this is the first study to evaluate whether the NLR can predict outcomes after first-line, second-line, or third-line capecitabine monotherapy among patients with metastatic or recurrent breast cancer. The results revealed that an NLR of <3 independently predicted better PFS but not better OS. Therefore, we cannot conclude that the NLR is useful for predicting OS among patients who receive capecitabine for metastatic or recurrent breast cancer. Additional research is needed to identify prognostic factors that can guide the selection of eribulin or capecitabine treatment in this setting.

## Supplementary Information

**Additional file 1.** Patient characteristics excluding duplicate cases.

**Additional file 2.** Univariate analyses of overall response rate.

**Additional file 3.** Univariate analyses of progression-free survival.

## Data Availability

To protect patient privacy, detailed patient records are not available. All relevant data are included in the manuscript and its associated files.

## Abbreviations

ALC: absolute lymphocyte count
CR: complete response
CRP: C-reactive protein
ECOG-PS: Eastern Cooperative Oncology Group-performance status
ER: oestrogen receptor
HER2: human epidermal growth factor receptor 2
HR: hormone receptor
LDH: lactate dehydrogenase
LMR: lymphocyte-to-monocyte ratio
NA: not available
NLR: neutrophil-to-lymphocyte ratio
ORR: overall response rate
OS: overall survival
PD: progressive disease
PFS: progression-free survival
PgR: progesterone receptor
PLR: platelet-to-lymphocyte ratio
PR: partial response
SD: stable disease
