# Supplementary material for: Neutrophil-to-lymphocyte ratio as a prognostic factor for patients with metastatic or recurrent breast cancer treated using capecitabine: a retrospective study
Source: BMC Cancer. 2022 Jan 14;22:64. doi: 10.1186/s12885-021-09112-9 (PMC8759263; doi:10.1186/s12885-021-09112-9)
Supplement: Supplementary file 1 — Additional file 1. Patient characteristics excluding duplicate cases. [file 12885_2021_9112_MOESM1_ESM.docx]

**Additional file 1. Patient characteristics excluding duplicate cases**

|  |  | Eribulin  (n=49) | Capecitabine  (n=37) | *p* |
| --- | --- | --- | --- | --- |
| Age in years, n (%) | Median (range) | 52 (30–70) | 56 (36–74) | - |
|  | ≥60 | 14 (29) | 15 (41) | 0.26 |
|  | <60 | 35 (71) | 22 (59) |  |
| Sex, n (%) | Male | 0 (0) | 0 (0) | - |
|  | Female | 49 (100) | 37 (100) |  |
| ECOG-PS, n (%) | 0 | 33 (67) | 21 (57) | 0.36 |
|  | 1 | 16 (33) | 15 (41) |  |
|  | 2 | 0 (0) | 1 (3) |  |
| HR status, n (%) | Positive | 31 (63) | 30 (81) | 0.09 |
|  | Negative | 18 (37) | 7 (19) |  |
| ER status, n (%) | Positive | 31 (63) | 28 (76) | 0.25 |
|  | Negative | 18 (37) | 9 (24) |  |
| PgR status, (%) | Positive | 25 (51) | 22 (59) | 0.37 |
|  | Negative | 24 (49) | 13 (35) |  |
|  | NA | 0 (0) | 2 (5) | - |
| HER2 status, n (%) | Positive | 4 (8) | 1 (3) | 0.39 |
|  | Negative | 45 (92) | 35 (95) |  |
|  | NA | 0 (0) | 1 (3) | - |
| Triple-negative, n (%) | | 15 (31) | 7 (19) | 0.32 |
| Surgical history, n (%) | Positive | 42 (86) | 31 (84) | 1.00 |
|  | Negative | 7 (14) | 6 (16) |  |
| Neoadjuvant/adjuvant  chemotherapy, n (%) | Positive | 36 (73) | 23 (62) | 0.35 |
|  | Negative | 13 (27) | 14 (38) |  |
| Previous hormone therapy, n (%) | Positive | 27 (55) | 33 (89) | **0.001** |
|  | Negative | 22 (45) | 4 (11) |  |
| Previous anthracycline,  n (%) | Positive | 46 (94) | 30 (81) | 0.09 |
|  | Negative | 3 (6) | 7 (19) |  |
| Previous taxane, n (%) | Positive | 48 (98) | 33 (89) | 0.16 |
|  | Negative | 1 (2) | 4 (11) |  |
| Previous chemotherapy regimens, n (%) | 0 | 9 (18) | 4 (11) | 0.63 |
|  | 1 | 17 (35) | 14 (38) |  |
|  | 2 | 23 (47) | 19 (51) |  |
| Response, n (%) | PR | 3 (6) | 6 (16) | **0.005** |
|  | SD | 22 (45) | 25 (68) |  |
|  | PD | 24 (49) | 6 (16) |  |
| Albumin, n (%) | ≥4.1 g/dL | 33 (67) | 23 (62) | 0.65 |
|  | <4.1 g/dL | 16 (33) | 14 (38) |  |
| LDH, n (%) | <222 U/L | 17 (35) | 24 (65) | **0.009** |
|  | ≥222 U/L | 32 (65) | 13 (35) |  |
| CRP, n (%) | <0.15 mg/dL | 22 (45) | 22 (59) | 0.20 |
|  | ≥0.15 mg/dL | 27 (55) | 15 (41) |  |
| NLR, n (%) | <3 | 32 (65) | 27 (73) | 0.49 |
|  | ≥3 | 17 (35) | 10 (27) |  |
| ALC, n (%) | ≥1,500/µL | 15 (31) | 17 (46) | 0.18 |
|  | <1,500/µL | 34 (69) | 20 (54) |  |
| LMR, n (%) | ≥5 | 20 (41) | 22 (59) | 0.13 |
|  | <5 | 29 (59) | 15 (41) |  |
| PLR, n (%) | <250 | 34 (69) | 32 (86) | 0.08 |
|  | ≥250 | 15 (31) | 5 (14) |  |

ALC, absolute lymphocyte counts; CRP, C-reactive protein; ECOG-PS, Eastern Cooperative Oncology Group-performance status; ER, oestrogen receptor; HER2, human epidermal growth factor receptor 2; HR, hormone receptor; LDH, lactate dehydrogenase; LMR, lymphocyte-to-monocyte ratio; NA, not available; NLR, neutrophil-to-lymphocyte ratio; PD, progressive disease; PgR, progesterone receptor; PLR, platelet-to-lymphocyte ratio; PR, partial response; SD, stable disease.
