# Supplementary material for: Neutrophil-to-lymphocyte ratio as a prognostic factor for patients with metastatic or recurrent breast cancer treated using capecitabine: a retrospective study
Source: BMC Cancer. 2022 Jan 14;22:64. doi: 10.1186/s12885-021-09112-9 (PMC8759263; doi:10.1186/s12885-021-09112-9)
Supplement: Supplementary file 2 — Additional file 2. Univariate analyses of overall response rate. [file 12885_2021_9112_MOESM2_ESM.docx]

**Additional file 2. Univariate analyses of overall response rate**

|  | Eribulin (N=91) | | | Capecitabine (N=79) | | |
| --- | --- | --- | --- | --- | --- | --- |
|  | CR/PR  n=14 (%) | SD/PD  n=77 (%) | *p* | CR/PR  n=16 (%) | SD/PD  n=63 (%) | *p* |
| HR+ | **14 (100)** | **57 (74)** | **0.034** | 15 (94) | 55 (87) | 0.68 |
| ER＋ | **14 (100)** | **54 (70)** | **0.018** | 14 (88) | 51 (81) | 0.72 |
| PgR+ | 11 (79) | 44 (57) | 0.15 | 10 (63) | 42 (69) | 0.77 |
| HER2- | 14 (100) | 71 (92) | 1.0 | 16 (100) | 59 (94) | 1.0 |
| Triple-negative | 0 (0) | 17 (22) | 0.06 | 1 (6) | 8 (13) | 0.68 |
| Surgical history | 13 (93) | 70 (91) | 1.0 | 13 (81) | 59 (94) | 0.14 |
| Neoadjuvant/adjuvant chemotherapy | 13 (93) | 63 (82) | 0.45 | 13 (81) | 50 (79) | 1.0 |
| Previous hormone therapy | 11 (79) | 52 (68) | 0.54 | 15 (94) | 57 (90) | 1.0 |
| Previous anthracycline | 13 (93) | 72 (94) | 1.0 | 14 (88) | 53 (84) | 1.0 |
| Previous taxane | 14 (100) | 74 (96) | 1.0 | 16 (100) | 57 (90) | 0.34 |
| Albumin ≥4.1 g/dL | 8 (57) | 47 (61) | 0.78 | 9 (56) | 42 (67) | 0.56 |
| Age ≥60 years | 6 (43) | 28 (36) | 0.77 | 8 (50) | 27 (43) | 0.78 |
| LDH <222 U/L | 7 (50) | 31 (40) | 0.56 | 10 (63) | 38 (60) | 1.0 |
| CRP <0.15 mg/dL | 8 (57) | 31 (40) | 0.26 | 8 (50) | 38 (60) | 0.57 |
| NLR <3 | 12 (86) | 53 (69) | 0.33 | **15 (94)** | **41 (65)** | **0.03** |
| ALC ≥1,500/µL | 8 (57) | 23 (30) | 0.07 | 11 (69) | 25 (40) | 0.05 |
| LMR ≥5 | 4 (29) | 32 (42) | 0.55 | 12 (75) | 33 (52) | 0.16 |
| PLR <250 | 14 (100) | 59 (77) | 0.06 | 16 (100) | 52 (83) | 0.11 |

ALC, absolute lymphocyte count; CR, complete response; CRP, C-reactive protein; ER, oestrogen receptor; HER2, human epidermal growth factor receptor 2; HR, hormone receptor; LDH, lactate dehydrogenase; LMR, lymphocyte-to-monocyte ratio; NLR, neutrophil-to-lymphocyte ratio; PD, progressive disease; PgR, progesterone receptor; PLR, platelet-to-lymphocyte ratio; PR, partial response; SD, stable disease.
