# Supplementary material for: Neutrophil-to-lymphocyte ratio as a prognostic factor for patients with metastatic or recurrent breast cancer treated using capecitabine: a retrospective study
Source: BMC Cancer. 2022 Jan 14;22:64. doi: 10.1186/s12885-021-09112-9 (PMC8759263; doi:10.1186/s12885-021-09112-9)
Supplement: Supplementary file 3 — Additional file 3. Univariate analyses of progression-free survival. [file 12885_2021_9112_MOESM3_ESM.docx]

**Additional file** **3. Univariate analyses of progression-free survival**

|  | Eribulin (n=91) | Capecitabine (n=79) |
| --- | --- | --- |
|  | *p* | *p* |
| HR+ | 0.08 | 0.4 |
| ER＋ | 0.32 | **0.029** |
| PgR+ | 0.06 | 0.37 |
| HER2- | 0.77 | 0.91 |
| Surgical history | **0.006** | **0.036** |
| Neoadjuvant/adjuvant chemotherapy | **0.045** | 0.27 |
| Previous hormone therapy | 0.11 | 0.66 |
| Previous anthracycline | 0.39 | 0.43 |
| Previous taxane | 0.41 | 0.11 |
| Albumin ≥4.1 g/dL | 0.91 | 0.63 |
| Age ≥60 years | 0.73 | 0.36 |
| LDH <222 U/L | 0.12 | 0.17 |
| CRP <0.15 mg/dL | 0.06 | 0.12 |
| NLR <3 | **0.011** | **0.011** |
| ALC ≥1,500/µL | 0.14 | **0.013** |
| LMR ≥5 | 0.23 | **0.001** |
| PLR <250 | **0.001** | **0.037** |

ALC, absolute lymphocyte count; CRP, C-reactive protein; ER, oestrogen receptor; HER2, human epidermal growth factor receptor 2; HR, hormone receptor; LDH, lactate dehydrogenase; LMR, lymphocyte-to-monocyte ratio; NLR, neutrophil-to-lymphocyte ratio; PgR, progesterone receptor; PLR, platelet-to-lymphocyte ratio.
